# Volcanic soil gas ^4^He/CO_2_ ratio: a useful geochemical tool for real-time eruption forecasting

**DOI:** 10.1038/s41598-024-57666-y

**Published:** 2024-04-05

**Authors:** Nemesio M. Pérez, Eleazar Padrón, Gladys Melián, Pedro A. Hernández, German Padilla, José Barrancos, Fátima Rodríguez, Luca D’Auria, David Calvo

**Affiliations:** 1 Instituto Volcanológico de Canarias (INVOLCAN), 38400 Puerto de la Cruz, Tenerife, Canary Islands Spain; 2 Environmental Research Division, ITER, 38600 Granadilla de Abona, Tenerife, Canary Islands Spain

**Keywords:** Volcanology, Geochemistry

## Abstract

At many dormant volcanoes, magmatic gases are not channeled through preferential degassing routes as fumaroles and only percolate through the flanks of the volcano in a diffuse way. This type of volcanic gas emission provides valuable information, even though the soil matrix contains an important atmospheric component. This study aimed to demonstrate that chemical ratios such as He/CO_2_ in soil gases provide excellent information on the evolution of volcanic unrest episodes and help forecast the volcanic eruption onset. Before and during the occurrence of the October 2011–March 2012 submarine of El Hierro, Canary Islands, more than 8500 soil He analyses and diffuse CO_2_ emission measurements were performed. The results show that the soil He/CO_2_ emission ratio began increasing drastically one month before eruption onset, reaching the maximum value 10 days before. During the eruptive period, this ratio also showed a maximum value several days before the period with the highest magma emission rate. The He/CO_2_ ratio was also helpful in forecasting the eruption onset. We demonstrate that this tool can be applied in real-time during volcanic emergencies. Our results also encourage a reevaluation of the global He emission from the subaerial volcanism.

## Introduction

Large amounts of volcanic gases are released by volcanoes to the atmosphere through soil or regolith, regardless of whether the volcanoes are active or quiescent^1–7^. Magmatic gases are released not only through preferential degassing routes as fumaroles and tectonic discontinuities but also percolate through the volcano’s porous flank and are released to the atmosphere in diffuse degassing from the soils^3,7–9^. This degassing type represents an important mechanism to dissipate energy at volcanoes^10^ and significantly contributes to global volcanic degassing^5^. Diffuse volcanic degassing disturbs the original biogenic and atmospheric-sourced chemical composition of soil gases at the surface environment of the volcano, generating enrichments of CO_2_, He and other gases. In the last 25 years, there has been considerable interest in the study of diffuse degassing as a powerful tool in volcano monitoring programs, particularly in those volcanic areas lacking visible gas emissions (plumes, fumaroles, hot springs, etc.)^2–4,8–11^. In addition to remote sensing techniques and permanent instrumental networks, soil gas emission surveys are one of the few geochemical methods that can be safely used during periods of volcanic unrest. They comprise direct measurements of degassing fluxes and/or the collection of soil gases at a certain depth using metallic probes and storage in vials for later laboratory analysis. Some of the most studied gases in soil degassing studies are He and CO_2_ because both species have similar low solubility in silicate melts at low pressures^12^ and are considered good geochemical tracers of magmatic activity^3,4^. However, their movement through the crust towards the surface is very different once they are exsolved from the silicate melts. While CO_2_, as a reactive gas, is affected by the occurrence of interfering processes^13^ (gas scrubbing by ground-waters and interaction with rocks, decarbonatation processes, biogenic production, etc.). He is chemically inert, radioactively stable, non-biogenic, highly mobile, and relatively insoluble in water^4^. These properties minimize the interaction of this noble gas with the surrounding rocks or fluids during its ascent towards the surface. Their geochemical differences yield a higher relative He/CO_2_ ratio in the fumarole gases than is actually present in the magma. Still, it decreases when the magma reservoir reaches enough pressure to generate incipient fracture systems approaching the eruption, thus releasing considerably more of the magma volatiles^14^. Volcanologists have used this behavior to predict impending eruptive activity and magma dynamics^14,15^, to detect the increase in magmatic gas supply, magmatic fluid injections in hydrothermal systems and episodes of magma ascent and depressurization^16–18^, to locate different magma reservoirs feeding the same volcanic system^19^, to identify spatial heterogeneities in the boiling of deep hydrothermal reservoirs^20^, to study active and inactive faults connected with deep volcanic plumbing system^21^ and to constrain the amount of geothermal CO_2_ retained in the bedrock between the hydrothermal reservoir and the surface^22^. However, the He/CO_2_ ratio present in the soil gases that are being released diffusively by big portions of the volcanic edifice has never been studied intensively during a real volcanic unrest.

In this work we present a detailed study of He emission data performed during the recent submarine eruption at El Hierro (Canary Islands) that started on 12 October 2011, allowing us to estimate the quasi-daily diffusive He emission rate and the calculation of the diffuse ^4^He/CO_2_ (hereinafter He/CO_2_) emission ratio of the entire emerged part of the island during the volcanic unrest. The main goal of this work is to study the evolution of the He/CO_2_ ratio of the gases released diffusively through the surficial environment of the soils during a volcanic unrest across all the surface of the volcanic system. To achieve this objective, we used values of diffuse CO_2_ and He already published^4,23^ and completed He emission time series with 94 new emission values. Following the original Failure Forecast Method^30–32^, we propose a novel approach that considers experimental errors in the measurements and the modeling through statistical bootstrapping^35^ to forecast a major geological event (volcanic eruption) with the temporal variation of the He/CO_2_ ratio of the gases released through the soils of El Hierro island.

### The 2011–2012 submarine volcanic eruption at El Hierro

**Figure 1:**
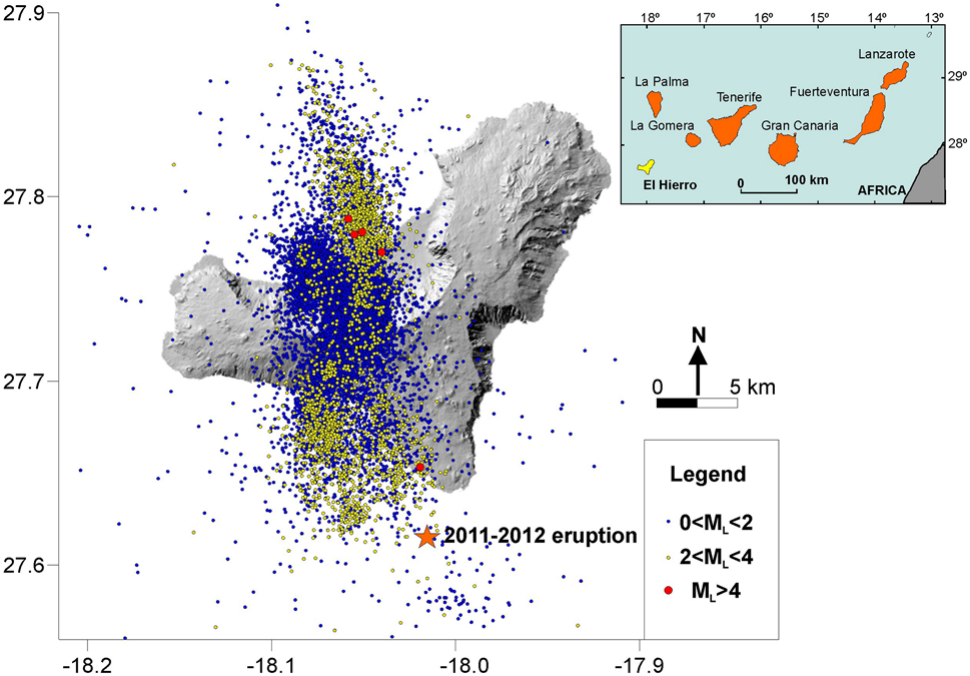
Geographic location of El Hierro Island on the Canarian Archipelago and epicenters of the 12,480 seismic events recorded by the Spanish National Geographic Institute (http://www.ign.es) in the period 15 July 2011–15 April 2012 on El Hierro Island. Star indicates the location of the submarine eruption, which was visible on 12 October for the first time. The maps were generated using Surfer Vesion 8.00 (http://www.goldensoftware.com/) and CorelDraw X3 (http://www.coreldraw.com). The seismic catalog is available at https://www.ign.es/ign/layoutIn/sismoFormularioCatalogo.do.

El Hierro (278 km^2^), located at the southwesternmost end of the Canary Islands, is the youngest island of this east–west trending volcanic chain of seven islands, with the oldest subaerial rocks dated at 1.12 Ma^24^ (Fig. 1). In July 2011, an anomalous seismic activity caused by magma movement beneath El Hierro that generated new fractures and microfractures was detected. The seismic catalog of the volcanic unrest was obtained through a changing seismic network which damaged the quality of data^25^ and prevented to have a homogeneous earthquake catalog. After the occurrence of more than 11,000 seismic events (Fig. 1), the magmatic reactivation process resulted in a shallow submarine eruption about 2 km off the south coast of the small village of La Restinga in the southernmost part of El Hierro (Fig. 1) and confirmed during the afternoon of 12 October 2011. The eruption occurred with a substantial lack of shallow seismicity between 8 km depth and the surface, probably because the magma found a pre-existing fragile area, which would permit an aseismic migration toward the surface^25,26^. The eruption finished on 5 March 2012 and caused abrupt changes in the physical–chemical properties of seawater^27^. This volcanic unrest was the first one to be monitored from the beginning in the 600 years of historical volcanism in the Canary Islands. Although the eruption took place in the underwater environment, the subaerial portion of the island was affected by anomalously high diffuse, deep-seated degassing of CO_2_, H_2_S, He and ^222^Rn detected on land weeks before the start of the submarine eruption^4,23,28–30^.

## Results

**Figure 2:**
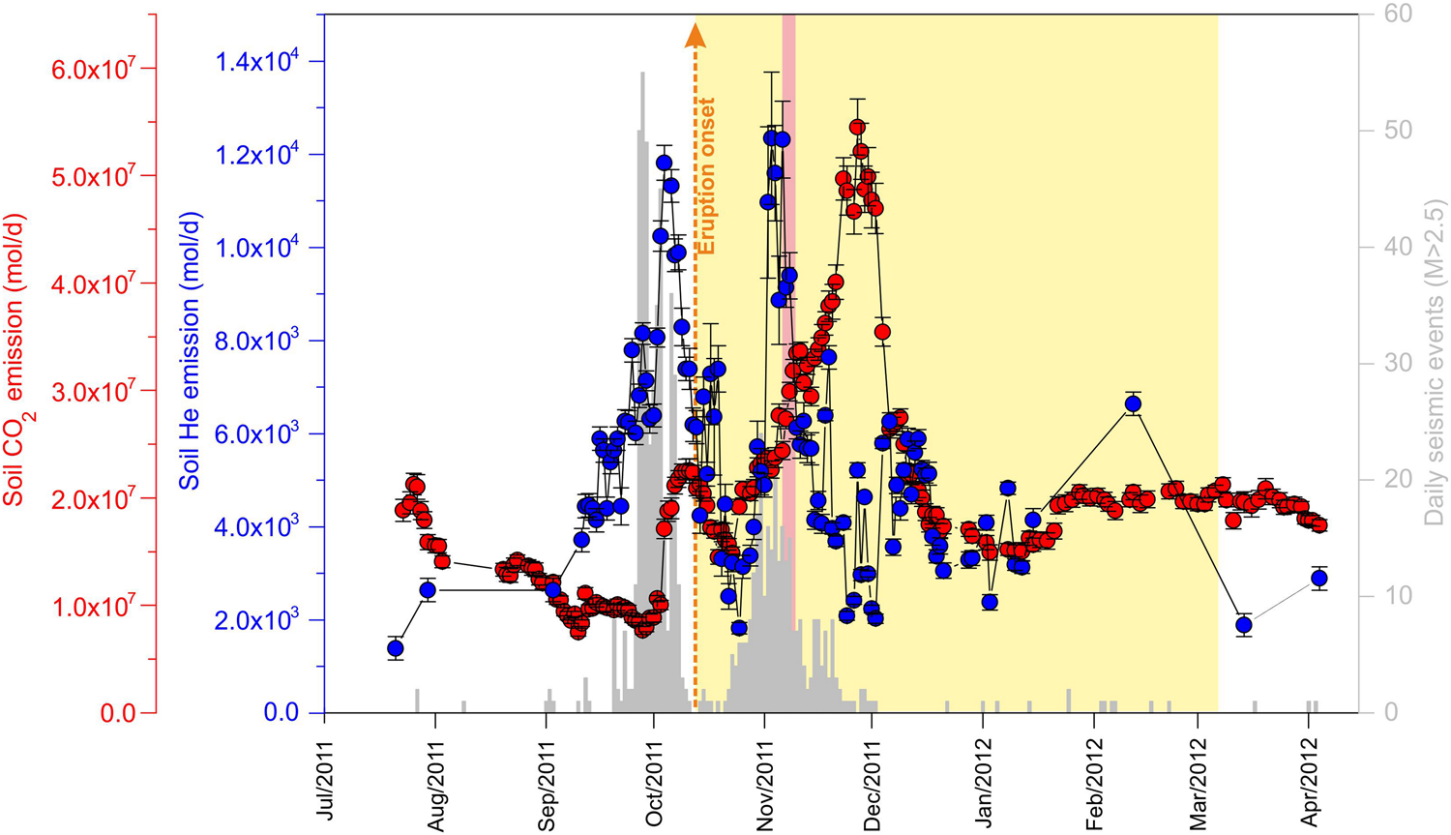
Temporal evolution of soil He (blue circles) and CO_2_^25^ (red circles) emissions and the number of seismic events with ML  > 2.5 (grey bars) recorded by the Spanish National Geographic Institute (http://www.ign.es) in the period 15 July 2011–15 April 2012 on El Hierro Island. The eruption onset is shown by a vertical orange arrow. The eruptive period is highlighted in yellow. The pink area highlights the period with the highest emission of lava^27^, between 5 and 10 November 2011, which coincided with the occurrence of large explosive “bubbles” at the sea surface, some potentially as high as 25 m on 5 November.

**Figure 3:**
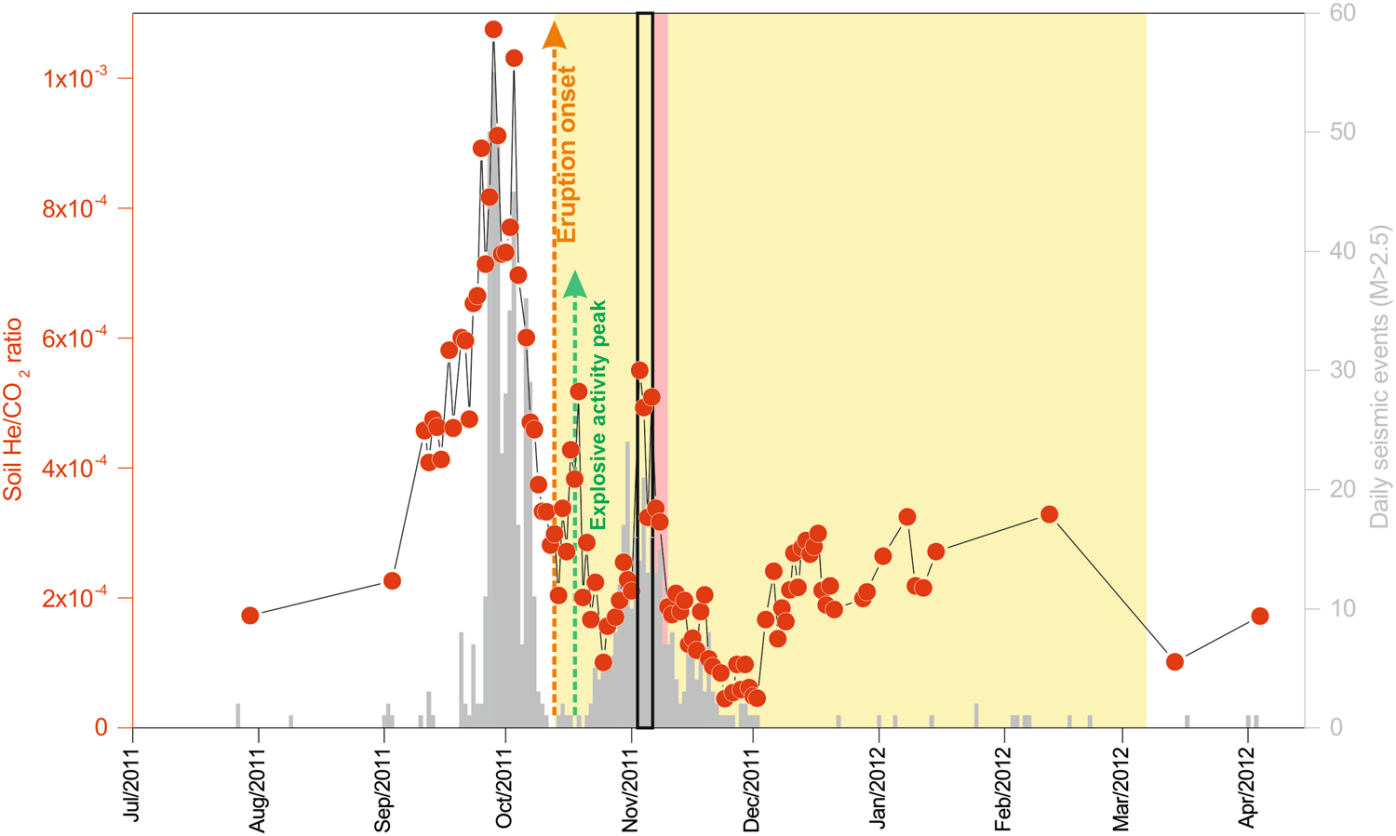
Temporal evolution of soil He/CO_2_ emission ratio and the number of seismic events with ML  > 2.5 (grey bars) recorded by the Spanish National Geographic Institute (http://www.ign.es) in the period 15 July 2011–15 April 2012 on El Hierro Island. The eruption onset is shown by a vertical orange arrow. The green arrow shows an important peak in the explosive activity during the early stages of the eruption^31^ (18 October 2011). The open black rectangle shows the He/CO_2_ emission peak (up to 5.5 × 10^−4^) between 3 and 4 November before the period with the highest magma emission rates^31^, highlighted by a pink area.

Before and during the occurrence of the October 2011–March 2012 submarine of El Hierro, Canary Islands, more than 8500 soil He analyses and diffuse CO_2_ emission measurements were performed. In this work, we completed the diffusive He emission rate time series already published^4^ with 94 new values (see methods section for details). Figure 2 depicts the temporal evolution of diffusive He and diffuse CO_2_ emissions of the entire island and the number of seismic events with M_L_ > 2.5 in the period 15 July 2011–15 April 2012. Before the eruption onset, diffusive He emission began to increase one month before the eruption, while diffuse CO_2_ emission started increasing only about one week in advance. This is reflected by a drastic increase in the He/CO_2_ emission ratio of the island, which reached the maximum value of the series (1.1 × 10^−3^) on 28 September 2011, two weeks before the eruption onset (Fig. 3). There was a difference of ~ 23 days between the start of the two anomalous He and CO_2_ emission rates (arrival at the surface of both gases). As can be observed in Fig. 3, the increase in the He/CO_2_ emission ratio also preceded increases in seismic activity during the volcanic unrest. Moreover, the He/CO_2_ emission ratio starts decreasing simultaneously with seismic activity, reaching a relative minimum at the eruption onset. The temporal analysis of the erupted volume indicated an intense explosive activity during the early stages of the eruption, showing an important peak on 18 October 2011 (green arrow in Fig. 3), six days after eruption onset^31^. This observation coincides with a new increase in the He/CO_2_ emission ratio, which reached a relative maximum only one day after this date (Fig. 3).

The period with the highest magma emission rate and hot hydrothermal fluids from the submarine vent (pink area in Fig. 2 and 3) took place between 5 and 10 November 2011^31^. It coincided with the occurrence of large explosive “bubbles” at the sea surface and during a new burst of seismicity that appeared off the northern coast of El Hierro since 16 October. We observed a significant He/CO_2_ emission peak (up to 5.5 × 10^−4^) between 3 and 4 November (open black rectangle in Fig. 3), some days before the mentioned period with the highest emission of lava (Fig. 3). The He peak again preceded in ~ 23 days the anomalous CO_2_ emission, which reached the maximum value on 27 November. Apart from the period when a significant increase in the number and magnitude of seismic events was recorded in November 2011, no spatial relationship was observed between the location of the epicenters of seismic activity and the higher diffusive He emission values.

The amount of He released diffusively through the surface environment of El Hierro during the study period (10 months), estimated by the integration of the He emission time series (Fig. 2), was ~ 9.7 tons. This value is slightly higher than those calculated by considering the estimated CO_2_ emission associated with the erupted volume (in the range 1.3–2.1 Tg CO_2_)^32^ and the He/CO_2_ mass ratio of the MORB (~ 4 × 10^−6^)^33^, that was in the range 5.2–8.4 tons.

Since gas emission rates and/or chemical variations, as the observed He/CO_2_ emission ratio, may be influenced by several factors associated with brittle fracture of the volcanic eruption, the drastic increase in this ratio, prior the eruption onset, can be considered as a geochemical precursory window to apply the Failure Forecast Method (FFM)^34–36^ and forecast the volcanic eruption. The FFM uses rock failure as a fundamental cause for much precursory activity, which can lead to volcanic eruptions or seismic events. We assume that the He/CO_2_ time series can be represented as a solution of the differential equation of the material failure empirical model. The extrapolation of the inverse rate plot (CO_2_/He) versus time to the time at which it is equal to zero, may be used to forecast the time of failure (eruption)^37^. In this work, we propose the application of the FFM in a probabilistic fashion (PFFM) by using the bootstrap method^38^, as explained in the methods section.

**Figure 4:**
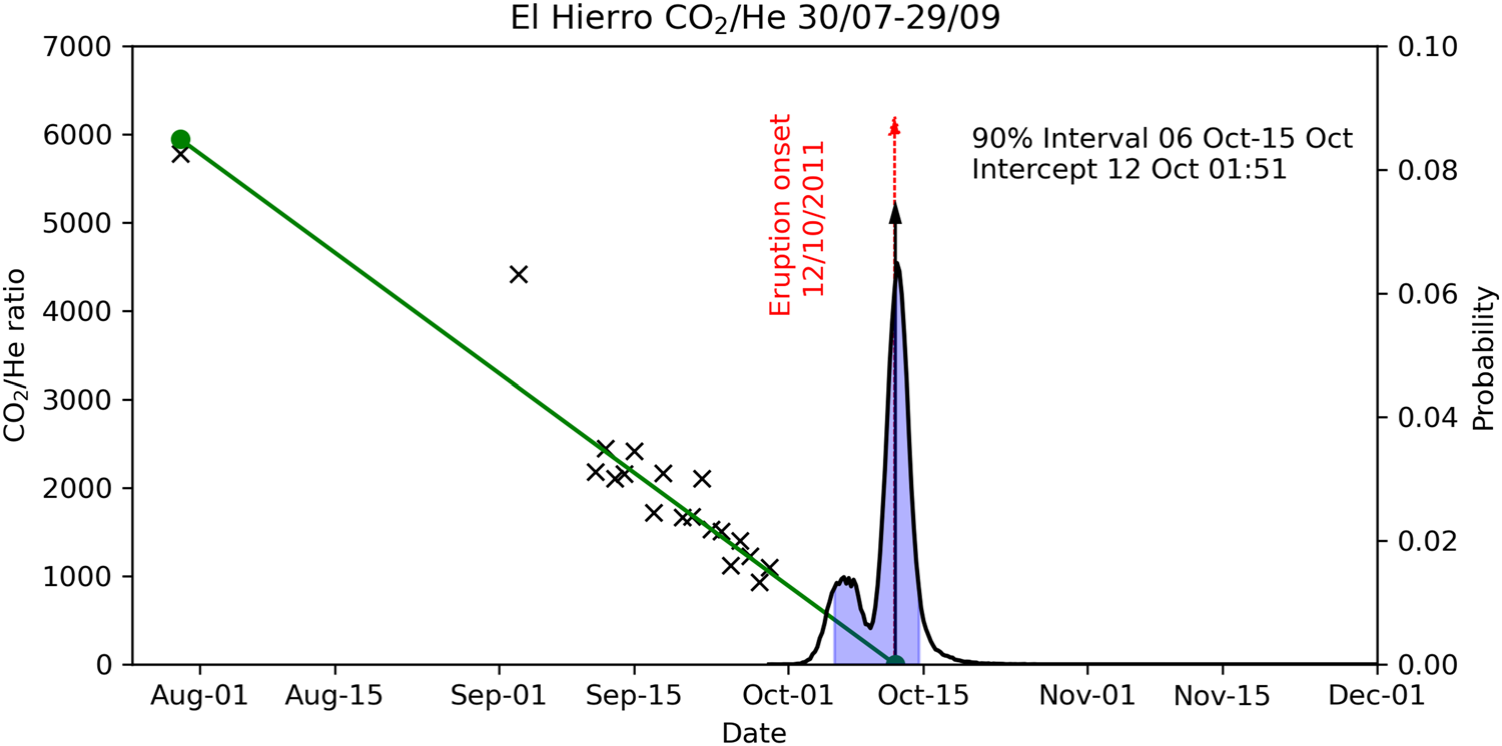
Example application of the PFFM for the day 29 September 2011. Black crosses indicate the CO_2_/He values measured until 29 September 2011. The blue curve marks the empirical p.d.f. obtained through bootstrapping. The red dashed arrow marks the eruption onset (12 October 2011), while the black arrow is the median of the p.d.f. The green line marks the best-fit straight line whose intercept with the x-axis coincides with the median of the p.d.f.

**Figure 5:**
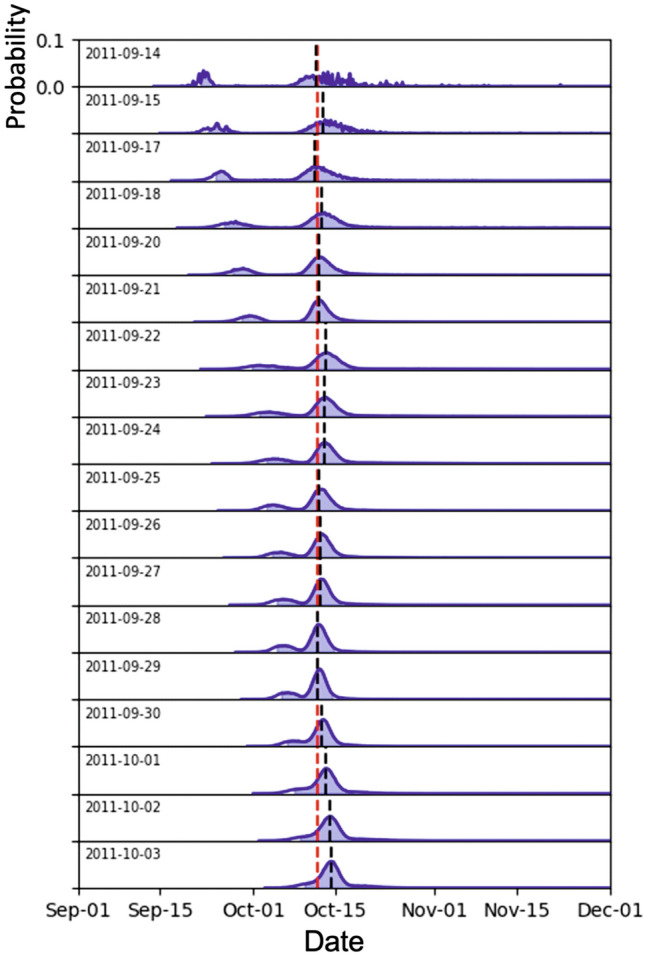
Demonstration of the real-time application of the PFFM to the CO_2_/He ratio. Each panel shows the application of the PFFM to each date. The blue curves mark the empirical p.d.f. for the date indicated in the upper left corner of each panel. The red dashed line marks the date of the eruption onset (12 September 2011), while the black dashed lines are the median of each p.d.f.

Figure 4 shows an example application of the PFFM on 29 September 2011, when the dataset consisted of 19 values. We can observe the bimodal probability density function (p.d.f.) of the intercept time, the 90% confidence interval evidenced in shades of light blue and the comparison between the median of the distribution (black arrow) and the onset of the eruption (red dashed arrow). In Fig. 5, we simulate the application of the PFFM in real-time during the month preceding the eruption. We observe the variation of the p.d.f. computed after each new sample is added to the dataset. The most striking observation is the closeness between the median and the actual eruption date for all the estimations and the fact that the actual eruption date always falls within the 90% confidence interval. The estimation stopped on 4 October 2011 because, after this date, the CO_2_/He ratio started increasing, making the application of the PFFM meaningless.

## Discussion

The new quasi-daily estimates of diffusive He emission values obtained with the proposed methodology cannot be considered as daily emission values throughout the entire island of El Hierro, but as a good approximation of a smoothed moving average of the real diffuse He emission time series. Keeping in mind that the He/CO_2_ emission data always preceded drastic changes in the seismic activity of El Hierro during the study period, the final time series shows an excellent fit with the histogram of the most energetic seismic events (M_L_ > 2.5), as is depicted in Fig. 3. The second peak involves an event that did not end in eruption and shows much less intensity than the first one. The observed second peak of He emission (Fig. 2), measured in the eruptive period, was likely caused by a combination of several factors: (i) the release of radiogenic helium produced by crustal deformation and fracturing supported by the isotopic composition of He in groundwaters^4^, (ii) an enhanced exolution of magmatic gases due to the decompression of the plumbing system after the eruption, and (iii) the degassing of magmatic volatiles from subsequent recharge of the shallower reservoir with fresh magmas^39^.

The occurrence of two different emission peaks for both He and CO_2_, with approximately the same delay between them of ~ 23 days, means a coherent behavior with the other geochemical properties and ascent times through the crust of both gases. The detailed time series of the He/CO_2_ emission ratio during the El Hierro 2011–2012 submarine eruption presented here demonstrate the importance of a high frequency monitoring, or even a continuous monitoring of this ratio through automatic stations in active volcanic regions, mainly at those areas without visible manifestations of volcanic fluid discharges. This continuous monitoring of the He/CO_2_ ratio in diffuse degassing represents a technological challenge, due to the very low levels of He emission that are expected to be measured. The discrete measurement of this ratio represents a new volcano monitoring method that may contain information from a wide area of the volcano (in our case, all the emerged parts of the volcanic edifice), thereby minimizing loss of information due to the inherent internal heterogeneity of the volcano.

The application of the PFFM to the He/CO_2_ ratio may be used successfully to forecast significant geological events such as the opening of the eruptive vent. Volcanic eruptions are preceded by increases in gas emission, that are used as precursors of incoming eruptions as they are linked to the magma movement at depth. Such magma rising from depth are the cause for both increases in gas emission and the failure of the surrounding rocks. Although the solubility of CO_2_ in magmatic melts is highly dependent on the type of magma, especially due to the content of certain cations, it can be stated that CO_2_ and He have similar low solubilities. For this reason, the process the modify in higher portion the He/CO_2_ ratio in the volcanic gases observed at the surface when the volcano is approaching a critical state (close to eruption) are the rock failure and the secondary processes that hinder the arrival of CO_2_ to the surface. The relationship between the brittle fractures caused by the magma rising and gas emission is evident as brittle fractures in the magma generates network of cracks that are progressively interconnected^40,41^. An explosion would occur by a rapid decompression when these networks of fractures filled by gas reach the surface. He/CO_2_ ratio in the volcanic degassing offers information of a major component of volcanic gases (CO_2_) and a non-reactive gas that moves easily within the crust toward the surface. Our results highlight the critical role that volcanic gases can play in understanding in real-time major volcanic events, even in the absence of visible manifestations of volcanic fluid discharges.

The differences between the observed He/CO_2_ ratio in the soil gases of El Hierro (in the range 4.5 × 10^−5^–1.1 × 10^−3^) and the MORB value (~ 4 × 10^−6^)^33^ can be attributed to a combination of two factors that result in an increase in the He/CO_2_ ratio in the shallow layers of the crust with respect to what is present in the MORB: the gas scrubbing processes that hinder the arrival of CO_2_ to the surface and the release of radiogenic helium produced by crustal deformation and fracturing. Previous results suggest that the second factor has a higher proportion in the second He/CO_2_ peak observed on 18 October 2011 (green arrow in Fig. 3), six days after eruption onset^4^.

The ^4^He emission estimated by the He/CO_2_ ratio of the MORB and the CO_2_ emitted during the eruptive process and presented above (5.2–8.4 tons) suggest an overestimation of the ^4^He emission released diffusively through the surface environment of El Hierro during the study period (10 months) calculated here (9.7 tons). However, and taking into consideration this overestimation, assuming a global ^4^He emission from the subaerial volcanism in ~ 80.8 tons/year^42^, our He emission result might represent a maximum value of roughly 12% of the annual He emission from the subaerial volcanism of the Earth. The He emission of El Hierro calculated by a pure diffusive mechanism are no unreasonable and suggest the need for a revaluation of the global emission values, taking into account the diffuse degassing of this gas at volcanic areas.

## Conclusions

This study shows that high diffuse He/CO_2_ emission measured in the soil environment preceded the 2011–2012 El Hierro submarine eruption. The methodology presented here enables the calculation of the diffuse He/CO_2_ emission ratio of an active volcanic area that represents one of the latest developments in the research of useful precursors to forecast volcanic eruptions. The methodology proposed in this work is applicable to any volcanic system, regardless of its geotectonic context. The use of this inexpensive geochemical tool that merge information of the big areas of the volcano, considering geochemical gaseous tracers with different geochemical behavior in their movement within the mantle and crust, helps to reduce the uncertainty in the prediction of major geological events as a volcanic eruption. The detailed time series of He/CO_2_ emission ratio during El Hierro 2011–2012 submarine eruption presented here demonstrate the importance of its continuous monitoring in active volcanic regions, mainly in areas without visible manifestations of volcanic fluid discharges. Even in a submarine eruption episode, where most of the volatiles due to magma degassing are released in the submerged part of the volcanic edifice, the soil gases in the subaerial part of the volcano offers a valuable information to study and monitor the eruptive process. Finally, the He emission of El Hierro calculated by a pure diffusive mechanism suggest the need for a revaluation of the global He emission values, taking into account the diffuse degassing of this gas at volcanic areas.

## Methods

### ***Determination of the He/CO***_***2***_*** from diffuse degassing***

The methods used to calculate the quasi-daily estimates of diffusive He emission from the reported data were the same as used for the detailed diffuse CO_2_ emission time series^25^. The diffuse CO_2_ emission was measured following the closed accumulation chamber method^43^. 601 diffuse CO_2_ efflux measurements sites were selected covering the whole subaerial environment of El Hierro island with site spacing about 400 m. To estimate an almost daily value of the diffuse emission rate of CO_2_, from the start of the seismic-volcanic crisis at El Hierro, it was daily computed by means of integrating the new last day 50–70 measurements to the previous 550–530 ones. This procedure allowed to recalculate the diffuse emission rate of CO_2_ on a quasi-daily basis, refreshing the measured sites with the newer values to obtain a more detailed time series of the diffuse CO_2_ emission rate from the start of the seismic-volcanic crisis at El Hierro^25^. The same methodology was used to complete time series of the diffusive He emission rate. This procedure allowed to recalculate the diffusive He emission on a quasi-daily basis, refreshing the measured sites with the newer values to obtain a more detailed He emission time series, and enables calculation of the diffuse He/CO_2_ emission ratio of the entire island during the volcanic unrest. We completed the diffusive He emission rate time series already published^4^ with 94 new values. Each CO_2_ and He emission rate value is the sum of the emission rate of 27,999 squared cells (100 × 100 m) constructed by sequential Gaussian simulation (sGs) provided by the *sgsim* program^44,45^. The CO_2_ and He effluxes data were used to construct spatial distribution maps using the sGs. The final maps were always constructed as an average of 100 equiprobable realizations performed over a grid of 27,999 squared cells following the experimental variogram model of the 601 sampling sites.

### Probabilistic failure forecast method (PFFM)

For each value of the CO_2_/He ratio (the inverse function of the observable He/CO_2_ variation) measured at a time t_k,_ we consider all the previous K measurements. Using a bootstrap method, we randomly resample this dataset 100.000 times, computing, for each random dataset, the intercept time of the linear least square fit with the x-axis. This allows for obtaining an empirical probability density function which we use for our analysis. Specifically, we use the 5th and 95th percentiles to define the 90% confidence range. Since most of the obtained p.d.f. highly multimodal, and with numerous outliers, we considered using the median instead of the maximum likelihood of the mean value to estimate the most reasonable forecast date.

## Data Availability

All data generated or analyzed during this study can be found at: https://zenodo.org/record/7466803#.Y6LDTtV_q5c.
